# Neutrophil-suppressive activity over T-cell proliferation and fungal clearance in a murine model of *Fonsecaea pedrosoi* infection

**DOI:** 10.1038/s41598-021-99847-z

**Published:** 2021-10-12

**Authors:** Leandro Carvalho Dantas Breda, Cristiane Naffah de Souza Breda, Gilberto Hideo Kaihami, José Roberto Fogaça de Almeida, Grasielle Pereira Jannuzzi, Lucas Gonçalves Ferreira, Sandro Rogério de Almeida

**Affiliations:** 1 Departamento de Análises Clínicas e Toxicológicas, Faculdade de Ciências Farmacêuticas, Universidade de São Paulo, Av Prof. Lineu Prestes, 580, São Paulo, SP 05508-000 Brazil; 2 Departamento de Imunologia, Instituto de Ciências Biomédicas, Universidade de São Paulo, São Paulo, Brazil; 3 Departamento de Bioquímica, Instituto de Química, Universidade de São Paulo, São Paulo, Brazil

**Keywords:** Innate immunity, Immunology, Infectious diseases, Fungal infection

## Abstract

Neutrophils are essential to control several fungal infections. These cells are commonly known for their pro-inflammatory activities. However, some studies have demonstrated the anti-inflammatory properties of neutrophils during certain infectious diseases, culminating in the inhibition of T cell proliferation. Chromoblastomycosis (CBM) is a deep and progressive mycosis that affects thousands of people worldwide. Although neutrophil infiltrates are observed in the lesion histopathology, the fungus can overtake the immune system response and destroy the host-infected tissue. The present study demonstrated that neutropenic animals had an increase in the IL-6 production in the spleen and liver, followed by a lower fungal burden in these organs up to 14 days of infection. Neutropenic animals also showed a lower *F. pedrosoi*-specific antibody production 14-days post infection and higher T-cell proliferation in the in vitro experiments after stimulation with *F. pedrosoi*-purified proteins. Taken together, our results suggest that the presence of regulatory neutrophils in the mouse model of *F. pedrosoi* infection could act favoring the spread of the fungus and the chronicity of the infection. These findings shed light on the CBM treatment, which might target neutrophil polarization as a new therapy approach to treat CBM lesions.

## Introduction

*Fonsecaea pedrosoi* is a dematiaceous fungus of the Herpotrichiellaceae family, known as the main agent of chromoblastomycosis (CBM) disease, a deep and chronic subcutaneous mycosis^1,2^. The CBM lesions are characterized by verrucous, erythematous papules, and atrophic lesions in the skin^3^. Similar to most fungal infections, the treatment of CBM is complex and usually demands multi-drug administration^4^ associated with heat/freezing therapy^5,6^ and, in some cases, surgery^7^. Moreover, costly and long-term treatments might result in high rates of disease relapse and treatment discontinuation. Therefore, understanding the host–pathogen interaction is crucial to develop new therapeutic tools for CBM treatment^8^. In the last decade, great effort was made to understand the host immune response in CBM infection (for a review in this topic see Ref.^8^). Skin biopsies from CBM patients show a characteristic external layer of fibrous tissue with an internal layer of thick granulomatous inflammatory tissue containing mainly macrophages and neutrophils^9–11^. Several studies have demonstrated that the macrophage is poorly activated in CBM and does not seem to play a crucial role in this disease^12–15^. However, the importance of neutrophils controlling fungal spread remains unclear. One of the leading studies demonstrating the neutrophil fungicidal activity against *F. pedrosoi* conidia was published in the 1990s by the Rozental group^16^. Recently, our group demonstrated that neutrophils eliminate *F. pedrosoi* conidia in vitro through TLR-2 and TLR-4-dependent phagocytosis and ROS production, whereas hyphal killing occurs through Neutrophil Extracellular Traps (NETs) released independently of ROS production and TLR-2/TLR-4 receptors^17^. Neutrophils—also named polymorphonuclear (PMN) cells—are granulocytes from the innate immune system, known to be essential in several fungal infections, including *Candida albicans*^18^, *Aspergillus fumigatus*^19^, *Cryptococcus neoformans*^20^, *Paracoccidioides brasiliensis*^21^, and *Sporothrix schenckii*^22^. Neutrophils are the first leukocytes to migrate from the bloodstream to the infection site to contain and eliminate foreign pathogens. Therefore, for a long time, they were considered highly pro-inflammatory cells with antimicrobial features such as phagocytosis, oxidative burst, degranulation, and NETs release. However, in 1987, Young and colleagues observed in mice bearing lung carcinoma a myeloid-lineage cell similar to neutrophils but with anti-inflammatory activities^23^. After 8 years, Pekarek and colleagues demonstrated that the elimination of granulocyte cells in vivo fostered the mice’s immune system by inhibiting tumor growth^24^. Over the past 15 years, the interest in these myeloid-lineage cells with suppressive features has increased, but the lack of a uniform nomenclature leads to misunderstandings by researchers. In 2007, the term PMN myeloid-derived suppressor cells (PMN-MDSCs) was suggested to designate these anti-inflammatory neutrophils^25^. Although this cell is usually studied in a cancer context, it is known to be related to other pathological conditions, such as autoimmune and infectious diseases. Recently, studies have demonstrated the PMN-MDSC accumulation in several fungal diseases such as *Cryptococcus neoformans*^26^, *Aspergillus fumigatus*^26,27^, and *Candida albicans*^28,29^.

Since CBM infection is a chronic fungal disease with chronic skin lesions characterized by severe neutrophils and macrophages infiltrated, we hypothesized whether these neutrophils were modulated by the fungus to a PMN-MDSC profile, favoring the fungal survival and the chronicity of the infection. So, we used a specific anti-granulocyte antibody (α-Gr1) to induce mouse transient neutropenia. We found that granulocyte-depleted animals had a lower fungal burden in the spleen and liver in the early and late phases of the infection. We also showed that neutropenic animals had higher T-cell proliferation and lower *F. pedrosoi* specific-antibody production. This pattern is usually observed in patients with mild disease, while lower T-cell response and greater antibody titers are associated with severe forms of the disease. Therefore, our results suggest that *F. pedrosoi* modulates neutrophils to an anti-inflammatory/PMN-MDSC profile with implications for host protection in the CBM infection.

## Material and methods

### Animals

The experimental protocols involving animals were previously approved by the Institutional Ethics Committee for Animal Care and Research at the School of Pharmaceutical Sciences (CEUA/FCF Protocol 474). The in vivo experiments were carried out following the recommendations of the ARRIVE Guidelines and the Guide for the Care and Use of Laboratory Animals of the National Institutes of Health. Briefly, male C57Bl/6 mice at 10–12 weeks of age were obtained from the Animal House Production and Experimentation Facility of the School of Pharmaceutical Sciences of the University of São Paulo. The animals were maintained in an SPF environment and housed in temperature-controlled rooms at 23–25 °C with food and water ad-libitum throughout the experiments. The euthanasia procedure was performed according to the American Veterinary Medical Association Guidelines for the Euthanasia of Animals (2020), using the overdose of ketamine and xylazine method. In this study, we used 4–5 animals per group and we performed the experiments in two independent days. These two experiments were compiled, unless otherwise stated. In this study, no strategies were used to blind or randomize our groups.

### Fungal strain and growth conditions

This study was conducted using *F. pedrosoi* strain CBS 271.37, which was previously isolated from a Brazilian patient diagnosed with CBM. The genome of *F. pedrosoi* CBS 271.37 is completely sequenced and this strain is commonly used to study *F. pedrosoi* infection due to their large infection capacity^30^. The *F. pedrosoi* was cultivated on a Sabouraud agar at 30 °C until the inoculum preparation. To avoid attenuation or loss of the fungal virulence, we inoculated some animals once a month and recovered the fungus from the mouse spleen and liver after 15–20 days of infection. The sample was kept on a Sabouraud agar until use (maximum of three passages). The fungi were transferred to 150 mL of potato dextrose broth (Difco, BD) and grown for 5 days at 30 °C under shaking. After this period, the inoculum was filtered through a 40 μM cell strainer. The conidial particles were obtained from the flow-through solution and centrifuged for 5 min in 300×*g* to precipitate the small hyphae and large conidia. The supernatant with the small conidia was collected and centrifuged for 10 min at 9000×*g* and then resuspended in 1 × PBS. The conidia concentration was determined by the Neubauer chamber counting.

### Peripheral blood neutrophil depletion

C57BL/6 animals at 8–12 weeks of age were treated with anti-Gr1 to deplete granulocytes (named depleted animals) or with isotype control anti-LTF2 (BioxCell^®^—named isotype control animals). Briefly, animals were intraperitoneally (i.p.) treated with different concentrations of anti-Gr1 (0–200 μg) on day − 1. After 24 h (day 0), peripheral blood was collected by tail snip, the red blood cells were lysed with a hypotonic solution, and a Cytospin slide was prepared. Leukocytes were stained using the Wright-Giemsa kit (InstantProv kit, New Prov^®^), and the differential leukocyte count was performed under conventional microscopy, in which 100 leukocytes were analyzed by their nuclear shape and morphology followed by the calculation of the neutrophil percentage.

**Figure 1:**
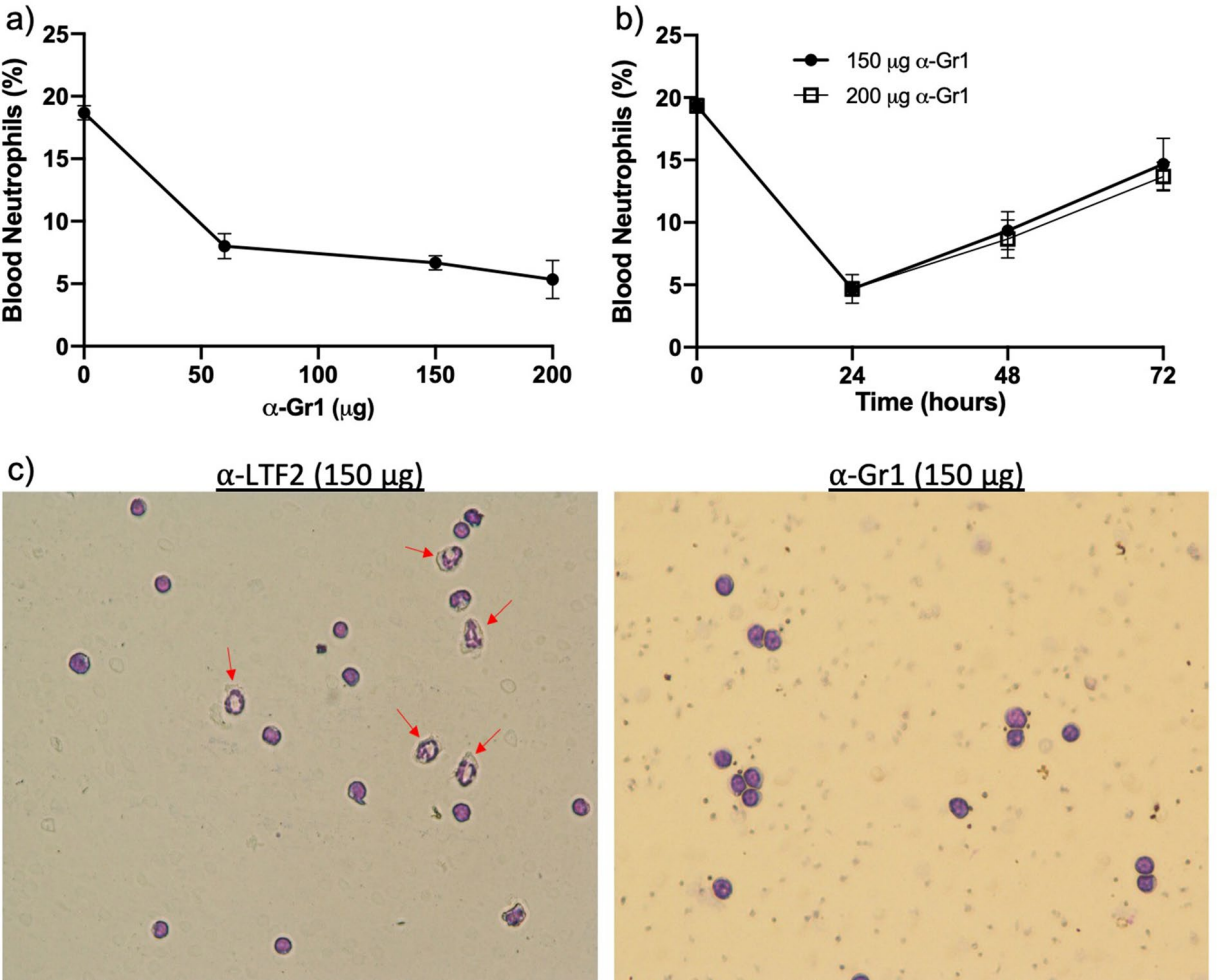
Peripheral blood neutrophil depletion: animals were treated intraperitoneally with different doses of α-Gr1 (0–200 μg). After 24 h, blood was collected by tail snip, and neutrophil depletion was analyzed under conventional microscopy (**a**,**c**). Neutrophil depletion was observed up to 72 h after a single dose of α-Gr1 (**b**). Data from three independent experiments (n = 2 animals per experiment) were compiled together. Red arrows show neutrophils—× 40 magnification (**c**).

The anti-Gr1 antibody was obtained from the supernatant of hybridoma culture (clone RB6-8C5). Approximately 1 × 10^7^ hybridoma cells were cultured in bioreactor flasks (Cell-Line Bioreactor—Argos Technologies) containing RPMI and 10% ultra-low IgG fetal bovine serum (ThermoFisher^®^), according to the bioreactor manufacturer’s instructions. After 5 days of cell culture, the supernatant was collected and frozen with 0.01% sodium azide until the IgG purification. The IgG purification was performed using the protein G column (Hitrap Protein G—GE Healthcare^®^) according to the manufacturer’s instructions. The samples were then placed into a dialysis membrane and kept in PBS solution for 24 h. The antibody concentration was determined using the NanoDrop equipment (ThermoFisher^®^). The anti-Gr1 functionality was first assessed by staining mouse neutrophils with secondary antibody anti-rat labeled with FITC followed by flow cytometry analysis (data not shown). Later, we tested the anti-Gr1 activity by analyzing the neutrophil depletion in the peripheral blood of murine animals after treatment with different concentrations of anti-Gr1 (Fig. 1).

### In vivo infection

The animals were i.p. infected with 5 × 10^7^ of *F. pedrosoi* conidia after 24 h of anti-Gr1 administration (day 0). They were euthanized after 1 and 2 days of infection to investigate the early phase of infection. For the late phase, animals received two extra doses of the antibody, one on day 3 and the other on day 7. After 14 days of infection, they were euthanized, and the spleen and liver were harvested to analyze fungal burden, cell populations, and cytokines production since these are the most commonly affected organs in this mouse model infection by *F. pedrosoi*. Briefly, the organs were harvested and smashed using a cell strainer (70 μM). An aliquot of the organ macerated was collected and seeded into Sabouraud agar for further colony-forming unit (CFU) analysis, and the remainder was centrifuged. After centrifugation, the supernatants were collected and frozen at − 80 °C for later cytokine measurement, while the cells were resuspended in 1 mL of PBS and placed over 3 mL Ficoll (1119 density) to isolate the leukocytes (top of the Ficoll layer) from the other tissue cells (bottom of the Ficoll layer). The leukocytes were collected from the top of the Ficoll layer and stained with proper antibodies for flow cytometry analysis (Suppl. Table 1). Cytokines from the organ supernatant were measured by the ELISA technique, according to the manufacturer's instructions (R&D Systems).

Because this work aimed to verify the role of neutrophils in a murine model of *F. pedrosoi* infection, we first analyzed the animals after 1 and 2 days of infection (early phase), since it is well-established that during this period, the main response of the immune system relies on the migration and activation of the innate cells, especially neutrophils^31,32^. Later, we intended to understand the consequences of the neutropenia in the adaptive immune system; therefore, we performed this analysis after 14 days of infection, when the adaptive immune system was fully activated.

### Lymphocyte proliferation assay

For the T cell proliferation evaluation, the spleen from the isotype control (α-LTF1) and neutropenic animals (α-Gr1) was harvested after 14 days of intraperitoneal infection with 5 × 10^7^ of *F. pedrosoi* conidia. The spleen was removed and the total cells were collected and stained with 10 μM CFSE (carboxyfluorescein diacetate succinimidyl ester), according to the manufacturer’s instructions (Molecular Probes). Next, 1 × 10^5^ cells/mL were plated and cultivated in supplemented R10 medium (RPMI containing 10% fetal bovine serum, 1% non-essential amino acids, and 1 mM sodium pyruvate) at 37 °C for 3 days. The non-stimulated cells were cultivated only with medium, whereas the stimulated cells were cultivated with medium containing 15 μg *F. pedrosoi* purified proteins (protein extraction protocol as described below). After this period, the cells were harvested and the T-cell proliferation was analyzed by flow cytometry (FACSCanto II—Becton Dickinson) by gating the CD3^+^ cells.

### Humoral immune response to *F. pedrosoi*

The host-specific antibody production against *F. pedrosoi* was analyzed by the Western blot technique. First, proteins from *F. pedrosoi* were extracted according to Almeida and colleagues with modifications^33^. Briefly, *F. pedrosoi* conidial and hyphal were washed three times in ultrapure water by centrifugation at 5000×*g* for 5 min (4 °C). The pellet was macerated with liquid nitrogen and a pestle until we get a slim powder. The proteins were suspended and vortexed for 5 min. The conidial and hyphal debris were removed by centrifugation (8000×*g*, 4 °C, 10 min). Protein concentrations were determined by the Bradford assay and the samples were kept at − 80 °C until use. We loaded an SDS-PAGE with the ladder marker and 10 μg *F. pedrosoi* proteins under reducing conditions. The proteins were transferred to nitrocellulose membranes, and nonspecific binding sites were blocked using 10% (w/v) dried milk in PBS-Tween (0.05%) for 2 h at RT. The membrane was then cut, so that each strip would contain two wells, one with the ladder marker and the other with the *F. pedrosoi* purified proteins. To assess the *F. pedrosoi*-specific antibody production by previously infected animals, each strip was incubated for 2 h with sera (diluted 1:200) from a single animal (isotype control or neutropenic). After washing, the strips were incubated with peroxidase-conjugated secondary antibodies for 60 min. Positive signals were detected by enhanced chemiluminescence (SuperSignal West Pico, Pierce). All strips were developed simultaneously and within the same exposure time (using the ImageQuant LAS 500—GE Healthcare^®^), so the densitometry of the bands could be determined (by the ImageJ program) and compared between the different groups of animals.

### Statistical analyses

Statistical analysis was performed using the GraphPad Prism^®^ program. Student *t*-test was used for the analyses between two groups with one variable. Data are expressed as the mean ± SEM, and the differences observed were considered significant when p < 0.05 (5%).

### Statement of ethics

The in vivo experiments were carried out following the recommendations of the ARRIVE Guidelines and the Guide for the Care and Use of Laboratory Animals of the National Institutes of Health. The experimental protocols involving animals were previously approved by the Institutional Ethics Committee for Animal Care and Research at the School of Pharmaceutical Sciences (CEUA/FCF Protocol 474).

## Results

### Anti-Gr1 treatment led to early severe neutropenia

First, we aimed to determine the necessary amount of α-Gr1 to induce mouse severe neutropenia. Thus, different amounts of anti-Gr1 antibodies were administrated to adjust the suitable dose of our model. Blood samples collected by tail snip showed a 50% decrease in circulating neutrophils after 24 h of antibody administration, even in the lowest dose (50 μg) (Fig. 1a–c). When increasing the antibody amount up to 200 μg, we enhanced the neutrophil depletion to approximately 75%. We also observed that a single shot of α-Gr1 maintained the neutrophil depletion around 50% after 48 h, but this depletion level decreased to approximately 25% after 72 h (Fig. 1b). We decided to use the lowest dose in our future set of experiments because no greater depletion was observed in animals treated with 200 μg α-Gr1 comparing to those who received 150 μg (Fig. 1b).

### Neutropenic animals showed a lower fungal burden in the early phase of *F. pedrosoi* infection

**Figure 2:**
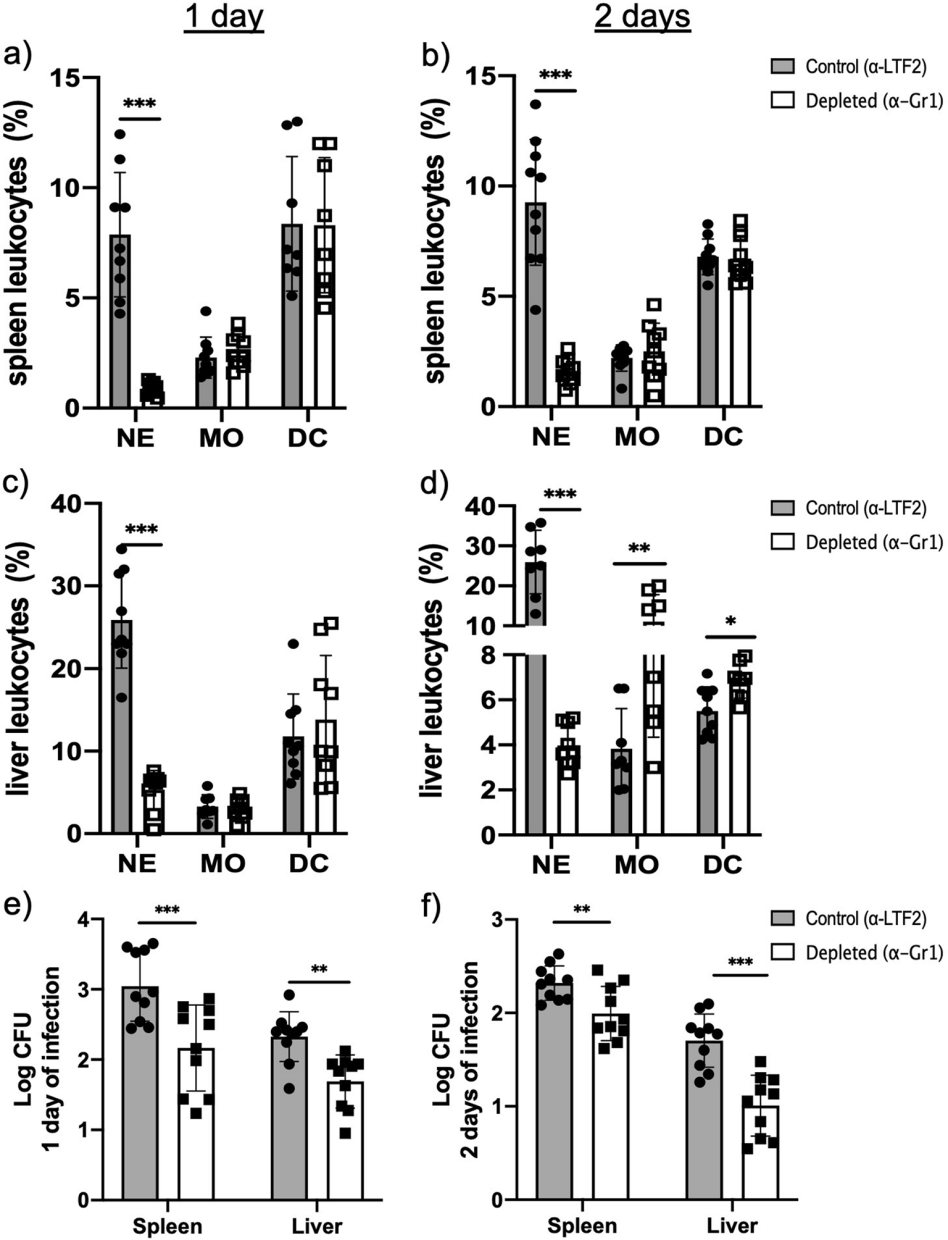
Leukocytes and fungal burden in the spleen and liver of isotype control and neutropenic animals infected with *F. pedrosoi*: Severe neutrophil depletion was observed after 1 day (**a**,**c**) and 2 days (**b**,**d**) of infection in animals treated with α-Gr1. An increase in the macrophage (MO) and dendritic cell (DC) population was observed in the liver after 2 days of infection (**d**). However, these changes were not observed in the spleen nor the liver after 1 day of infection (**a**–**c**). A lower fungal load was recovered from the spleen (**e**) and liver (**f**) of neutropenic animals after 1 and 2 days of infection. Results from two independent experiments (n = 4–5 animals per group and experiment) were compiled together. Values are expressed as the mean ± SD. *p < 0.05; **p < 0.01; ***p < 0.001, two-tailed unpaired Student *t*-test.

Once we defined the antibody dose to deplete the neutrophils, we treated the animals with 150 μg of α-Gr1 (or anti-LTF2, isotype control) on day − 1, followed by the infection with *F. pedrosoi* (day 0). We showed a great depletion of neutrophils in the spleen (Fig. 2a,b) and liver (Fig. 2c,d) of the animals after 1 and 2 days of infection. An increase number of macrophages (MO) and dendritic cells (DC) was also observed in the liver 2-days post infection (Fig. 2d); however, these changes were not present in the spleen nor liver of the 1 day-infected animals (Fig. 2a–c). Neutrophil-depleted animals showed a reduction in fungal burden in the spleen and liver after 1 and 2 days of infection (Fig. 2e,f), suggesting that the neutrophils have a PMN-MDSCs profile with an anti-inflammatory activity over the *F. pedrosoi* infection.

### Neutrophil-depleted animals have higher levels of IL-6 during the early phase of infection

**Figure 3:**
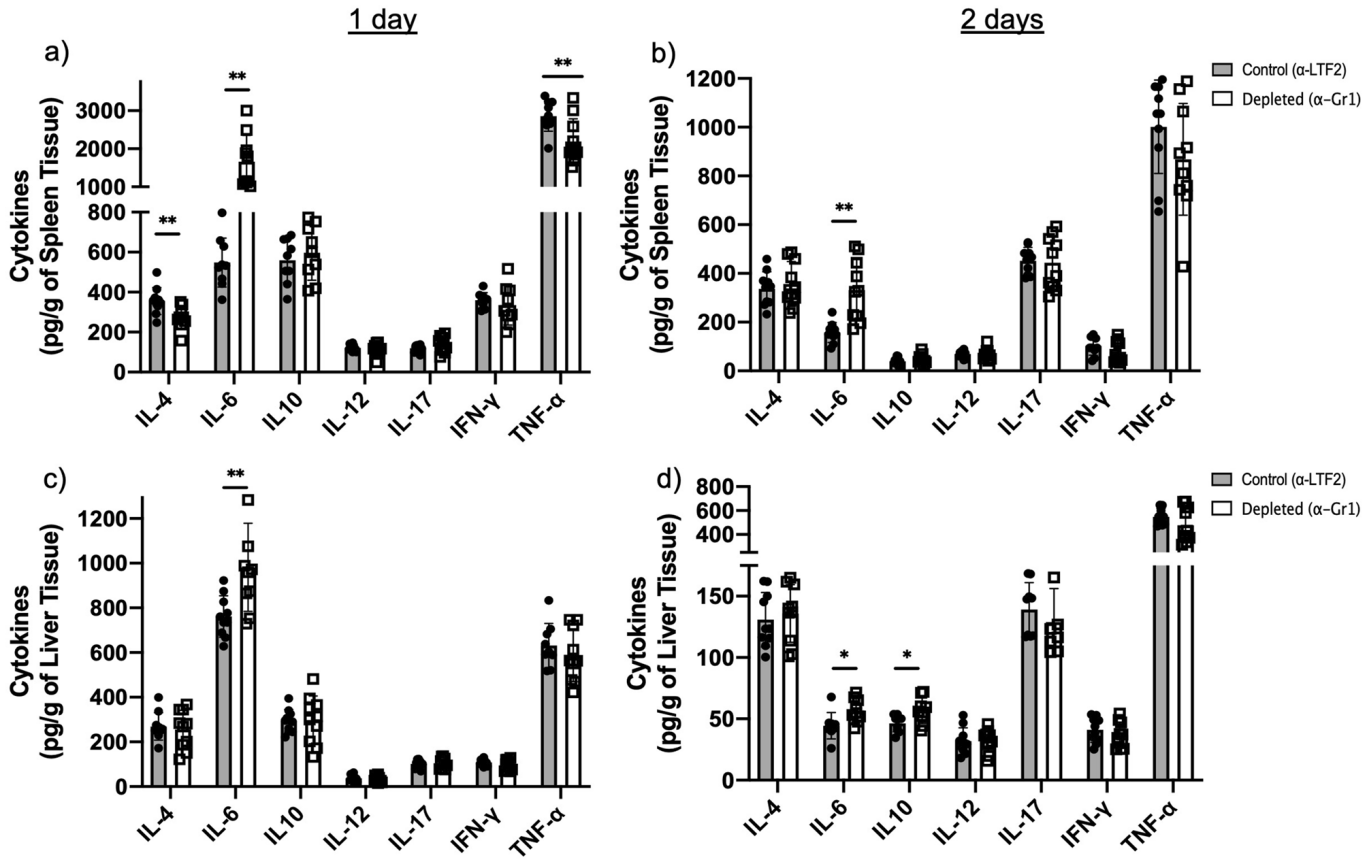
Cytokine production in the spleen and liver of *F. pedrosoi-*infected animals: We verified an increase in IL-6 production in the spleen and liver of neutropenic animals on day 1 (**a**,**c**) and day 2 of infection (**b**,**d**). A decrease in TNF-α in the spleen of neutropenic animals was observed after 1 day of infection (**a**) along with an increase of IL-10 in the liver after 2 days of infection (**d**). Results from two independent experiments (n = 4–5 animals per group and experiment) were compiled together. Values are expressed as the mean ± SD. *p < 0.05; **p < 0.01, two-tailed unpaired Student *t*-test.

When we assessed the cytokines production in the spleen and liver of the animals, we observed higher levels of IL-6 in neutropenic animals 1- and 2-days post infection (Fig. 3a–d) together with an increased level of IL-10 in the liver of these animals 2-days post infection (Fig. 3d). However, IL-4 and TNF-α levels were decreased in the spleen of neutropenic animals 1-day post infection (Fig. 3a). No differences in the IL-12, IL-17 and IFN-γ production was observed comparing the isotype control and neutropenic animals during the early phase of the infection.

### Neutropenic animals showed lower levels of B-cells

**Figure 4:**
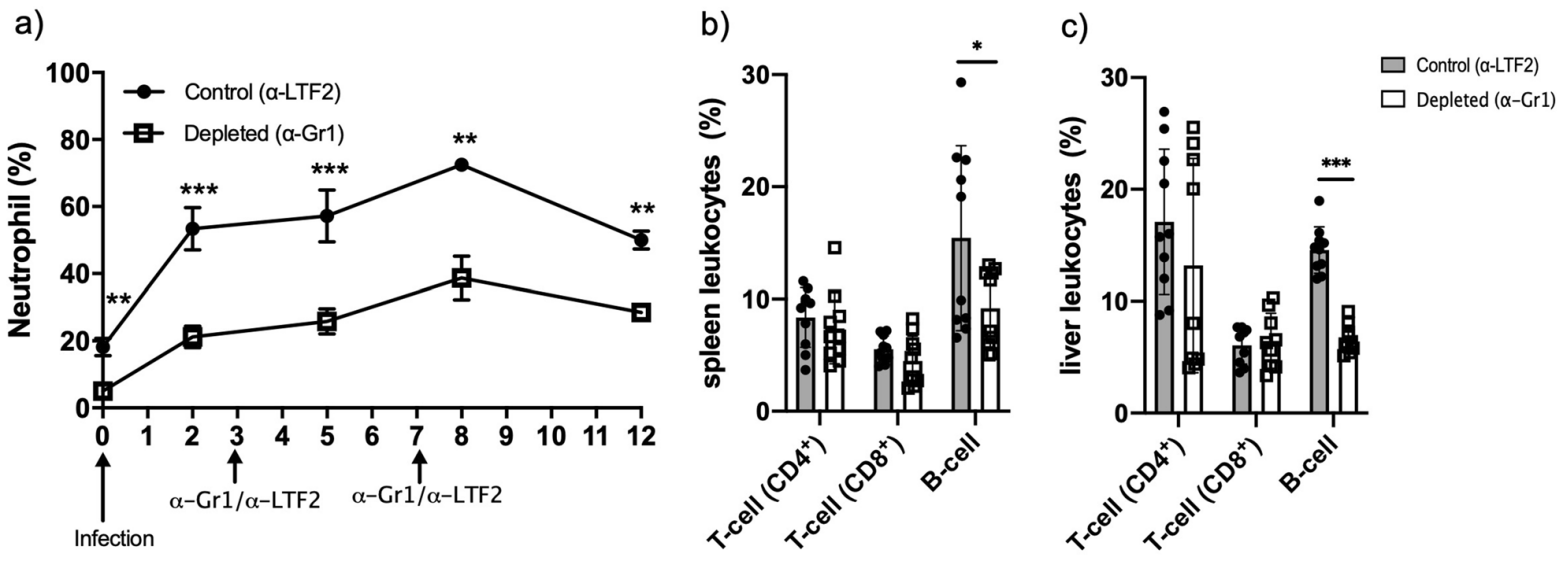
Neutrophil and lymphocytes analysis of isotype control and neutropenic animals after 14 days of *F. pedrosoi* infection: Peripheral blood neutrophil level was monitored for 12 days after *F. pedrosoi* infection (**a**). After 14 days of infection, T and B-cells from the spleen (**b**) and liver (**c**) were analyzed by flow cytometry according to the antibodies described in Suppl. Table 1. Results from two independent experiments (n = 4–5 animals per group and experiment) were compiled together. Values are expressed as the mean ± SD. *p < 0.05; **p < 0.01; ***p < 0.001, two-tailed unpaired Student *t*-test.

To better understand the influence of neutrophils over the adaptive immune response, a 14-day infection was induced in isotype control and neutropenic animals. Once we showed that a single shot of α-Gr1 kept the neutrophil depletion up to 72 h (Fig. 1), we included two extra doses to keep the neutrophil depletion for a longer period. Briefly, we performed the same protocol as described above and included one extra dose on day 3 and another on day 7. The neutrophil level was tracked by tail snip blood smear on days 2, 5, 8, and 12 after infection (Fig. 4a). Using the 14-day time-point, we aimed to analyze the lymphocytes population. We verified that neutropenic animals presented lower levels of B-lymphocytes with no difference in the T-lymphocyte populations (Fig. 4b,c).

### Neutrophil-depleted animals showed higher T-cell proliferation and a lower humoral response

**Figure 5:**
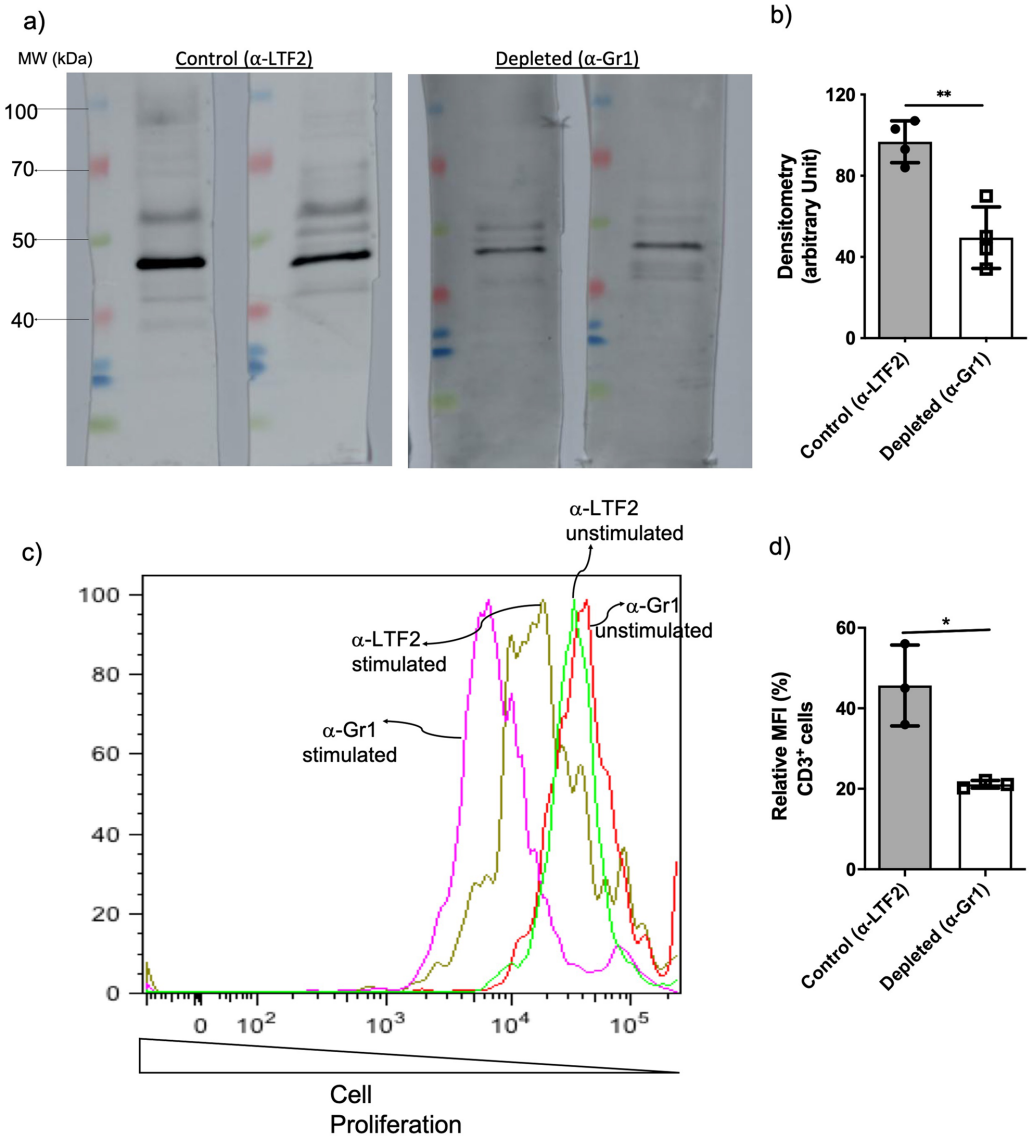
B and T-cells immune response after 14 days of infection with *F. pedrosoi*: B-cell response was analyzed by specific-antibody production against *F. pedrosoi* antigens by Western blot (**a**). The *F. pedrosoi* specific antibody production by isotype control and neutropenic animals was assessed by Western blot using the serum from previously infected animals against purified proteins of *F. pedrosoi*. Briefly, *F. pedrosoi* antigens were separated by SDS-PAGE electrophoresis and then transferred to the nitrocellulose membrane. Then, the membranes were cropped, and each membrane strip—containing the ladder and the *F. pedrosoi* purified proteins—were incubated with the sera of one animal—previously infected with *F. pedrosoi* (isotype control or neutrophil-depleted animal). All the membranes were developed simultaneously, therefore, with the same exposure time. Densitometry of Western blot bands confirmed the lower production of *F. pedrosoi* specific-antibodies in neutropenic animals (**b**). T-cells harvested from the spleen of neutropenic animals showed a higher capacity of in vitro proliferation (**c**,**d**). Data are expressed as the mean ± SD of three to four mice per group. The results are one representative of two independent experiments.

We next determined whether the presence of neutrophils was impairing the humoral and cellular immune response in *F. pedrosoi* infection. Besides the lower levels of B-cell (Fig. 4b,c), neutropenic animals also showed a decreased production of a specific antibody against *F. pedrosoi*, confirming the impairment of humoral response in these animals (Fig. 5a,b). However, assessing the T-cell function through the proliferation assay we verified a higher cellular immune response in neutropenic animals (Fig. 5c,d), suggesting the presence of PMN-MDSC in control animals impairing the T-cell proliferation.

### Neutropenic animals showed a better outcome during the late phase of the *F. pedrosoi* infection

**Figure 6:**
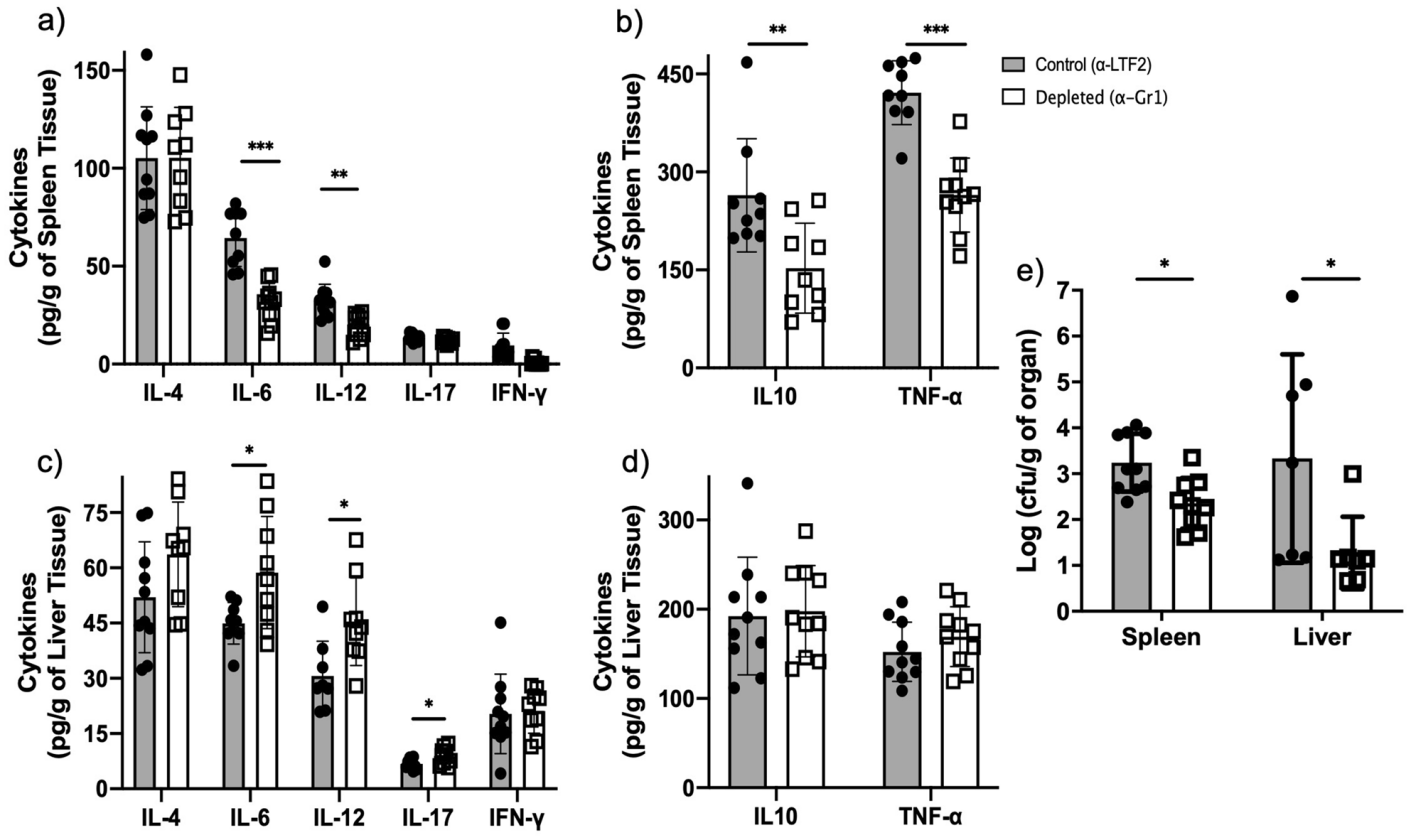
Cytokine and fungal load in the spleen and liver of animals after 14 days of infection with *F. pedrosoi*: We verified a decrease in IL-6, IL-10, IL-12, and TNF-α productions in the spleen of neutropenic animals (**a**,**b**) and an increase of IL-6, IL-12, and IL-17 productions in the liver of those animals (**c**,**d**). A lower fungal load was recovered from the spleen and liver of neutropenic animals after 14 days of infection. (**e**) Results from two independent experiments (n = 4–5 animals per group and experiment) were compiled together. Values are expressed as the mean ± SD. *p < 0.05; **p < 0.01, two-tailed unpaired Student *t*-test.

Having characterized the adaptive immune response in the neutropenic and control animals, we analyzed the cytokines production during the late phase of the infection. We verified lower concentrations of IL-6, IL-10, IL-12, and TNF-α in the spleen (Fig. 6a,b) but higher concentrations of IL-6, IL-12, and IL-17 in the liver of neutropenic animals (Fig. 6c,d). Moreover, in agreement with our previous findings of 1- and 2-days post infection, a lower fungal burden was also observed in the spleen and liver of neutropenic animals 14-days post infection, demonstrating a better clearance of *F. pedrosoi* in the absence of PMN-MDSC (Fig. 6e).

## Discussion

Fungal infections are known for their high complexity treatment. Several fungus species can evade the host immune system, leading to the chronicity of the disease. Chronic CBM skin wounds show a high rate of neutrophil and macrophage infiltrates, suggesting that the cell migration is not impaired in this disease^9–11^. While macrophages have a low fungicidal capacity in in vitro studies, probably because *F. pedrosoi* can inhibit nitric oxide production in these cells^14,15^, neutrophils show an important in vitro fungicidal capacity over *F. pedrosoi* conidia and hyphae^16,17^. However, the importance of macrophages and neutrophils in in vivo CBM infection is poorly understood. Since neutrophils with suppressor features were recently observed in *Cryptococcus neoformans*^26^, *Aspergillus fumigatus*^27^, and *Candida albicans*^28,29^ infections, we wondered whether *F. pedrosoi* could also modulate the neutrophils activities in vivo, leading to the inhibition of the host immune response and the chronicity of the infection. To answer this question, we induced transient neutropenia in the mouse by the α-Gr1 administration approach. We verified a significant decrease of neutrophils in the peripheral blood, spleen, and liver of those animals after 24 and 48 h of *F. pedrosoi* infection. The absence of neutrophils leads to a better clearance of the fungal infection (Fig. 2e,f), followed by higher IL-6 production in the spleen and liver after the first 48 h of infection (Fig. 3). This cytokine is generated in the infectious lesion by different cell types from the innate immune cells to mesenchymal cells, endothelial cells, fibroblasts, and others^34^. First described in the 1980s, IL-6 presents a pleiotropic effect on inflammation, immune response, and hematopoiesis^35^. At present, the role of IL-6 over granulopoiesis^36^ and PMN-MDSCs differentiation^37^ is well defined. Therefore, our results suggest that the *F. pedrosoi* modulates neutrophils’ activity to a PMN-MDSCs profile in the early phase of the host immune response through the stimulation of IL-6 production. Once these cells are depleted, a higher accumulation of IL-6 is seen at the lesion site of α-Gr1 treated animals. Similar results were observed in *Pneumococcus pneumoniae*^38^ and Trypanosoma *cruzi*^39^ infections, in which neutropenic animals showed lower pathogen load and higher levels of IL-6. Although the PMN-MDSCs are usually related to T-cell inhibition^40,41^, recent studies have demonstrated that these cells can also modulate the innate immune cells such as MO^42,43^, DC^44–46^, and Natural Killer (NK) Cells^27,47–49^. The relevance of these cells in in vivo CBM infection remains unclear. We believe *F. pedrosoi* stimulates PMN-MDSCs accumulation as an immune evasion mechanism, resulting in the impairment of the innate immunity activation and progression of the infection. However, a detailed study of the innate immune response in in vivo CBM infection is necessary to confirm this hypothesis.

Although MDSCs exert an important suppressor activity on innate immunity, the primary inhibitory activity occurs on adaptive immunity^50^. Here, we demonstrated an increased T-cell proliferation (Fig. 5c,d) and a lower B-cell population (Fig. 4b,c) followed by impaired *F. pedrosoi* specific-antibody production (Fig. 5a) by neutrophil-depleted animals. These findings suggest that the neutrophils infiltrate on the spleen of *F. pedrosoi* infected animals shows an anti-inflammatory profile (MDSCs profile), inhibiting the T-cell proliferation ex-vivo. The antibodies analysis from sera of infected animals identified two major groups of *F. pedrosoi* antigens, one with lower immunogenicity weighing around 54 kDa and a higher immunogenic group around 48 kDa. At least two previous studies had similar results, showing antibodies from CBM patients’ serum reacting to an *F. pedrosoi* antigen weighing around 54 kDa^51,52^. In addition to the 54 kDa antigens, Vidal and colleagues also observed a strong immune reaction to 66 kDa antigens^52^. We believe this difference might be due to the different protocols used for *F. pedrosoi*-antigen extraction or the species-specific humoral response of CBM infection. Previous studies showed that the host humoral response is not protective in human CBM infection and the severity of the disease is directly proportional to humoral response activation and indirectly proportional to the T-cell response activation^53–56^. Therefore, our findings corroborate these previous studies, demonstrating that PMN-MDSCs-depleted animals developed a mild infection—with lower spleen and liver fungal load (Fig. 6e)—associated with higher T-cell proliferation and lower antibody levels (Fig. 5). Although the cytokine profile did not follow a clear pattern, we believe that lower levels of IL-10 (Fig. 6b) in the spleen of neutropenic animals favor a higher rate of splenic T-cell proliferation ex vivo (Fig. 5)*,* once IL-10 is known to stimulate PMN-MDSCs activities such as inhibition of T-cell proliferation^42,57,58^. However, no cytokines were measured during the T-cell lymphoproliferative assay to confirm this hypothesis.

It is important to mention that like others experimental models, the murine model of *F. pedrosoi* infection has some limitations. This model does not mimic exactly the human disease. While the human disease is chronic and requires a long-lasting and complex treatment, the mouse can easily eliminate the infection after 25–30 days. Therefore, in order to assess the infectious/inflammatory process in our model we chose the 14-day time point after the infection (our late phase) to compare the fungal burden in the presence or absence of neutrophils.

Several studies have reported that the survival, differentiation, and immunosuppressive activity of MDSCs are affected by different Toll-like receptors (TLRs)^59^. Activation of TLR-4 by BCG^60^ and Immunomax^®^ immunostimulant^61^ decreased MDSCs frequency, whereas stimulation of TLR-7/8 by Imiquimod switched the anti-inflammatory profile of MDSC to a pro-inflammatory and antitumoral profile^62^. Therefore, TLRs-agonists arise as potential drugs for cancer treatment, being the Imiquimod recently approved by the Food and Drug Administration (FDA) for basal cell carcinoma treatment^63^. Although this is the first study demonstrating the presence of MDSCs in CBM infection, previous studies have successfully used TLR-4^64^ and TLR-7/8 agonists to treat murine CBM disease^65,66^. Several authors suggested that *F. pedrosoi* poorly activates the host immune system; therefore, the TLR-agonists are necessary to boost the host immune response and kill the infection. However, a high infiltration of neutrophils and macrophages is seen in patients’ wounds, suggesting that the host immune system is, at least, partially activated by the fungus. Thus, we believe that *F. pedrosoi* negatively regulates the host immune response stimulating the MDSCs differentiation and proliferation. The treatment with TLR-agonists would help the host immune system subverting these anti-inflammatory cells to a pro-inflammatory profile, favoring the resolution of the infection. Therefore, this work established a new field of study in CBM infection, demonstrating for the first time that *F. pedrosoi* stimulates PMN-MDSCs and inhibits host immune response leading to the chronicity of the infection. However, the main question still needs to be addressed: What is the mechanism used by *F. pedrosoi* to stimulate the proliferation and differentiation of PMN-MDSC?

## Supplementary Information

Supplementary Information.

## Data Availability

Data sharing do not apply to this article as no datasets were generated or analyzed during the current study.
