# Supplementary material for: Neutrophil-suppressive activity over T-cell proliferation and fungal clearance in a murine model of Fonsecaea pedrosoi infection
Source: Sci Rep. 2021 Oct 12;11:20220. doi: 10.1038/s41598-021-99847-z (PMC8511260; doi:10.1038/s41598-021-99847-z)
Supplement: Supplementary file 1 — Supplementary Information. [file 41598_2021_99847_MOESM1_ESM.pdf]

Supplementary Files:

Suppl. Table 1: Antibody panel used for flow cytometry analysis (antibody dilution 1:150)

Antibody	Catalog	Brand	Neutrophils	Macrophages	Dendritic Cells	T-cells	B-cells
CD45	557235	BD Biosc	+	+	+	+	+
CD3	100306	Biolegend	-	-	-	+	-
CD4	100511	Biolegend	-	-	-	+	-
CD8	553035	BD Biosc	-	-	-	+	-
CD11b	101205	Biolegend	+	+	-	-	-
CD11c	117318	Biolegend	-	-	+	-	-
CD19	557655	BD Biosc	-	-	-	-	+
F4/80	123110	Biolegend	-	+	-	-	-
CD45R	553091	BD Biosc	-	-	-	-	+
Ly6-G	127614	Biolegend	+	-	-	-	-
MHC-II	107628	Biolegend	-	-	+	-	-

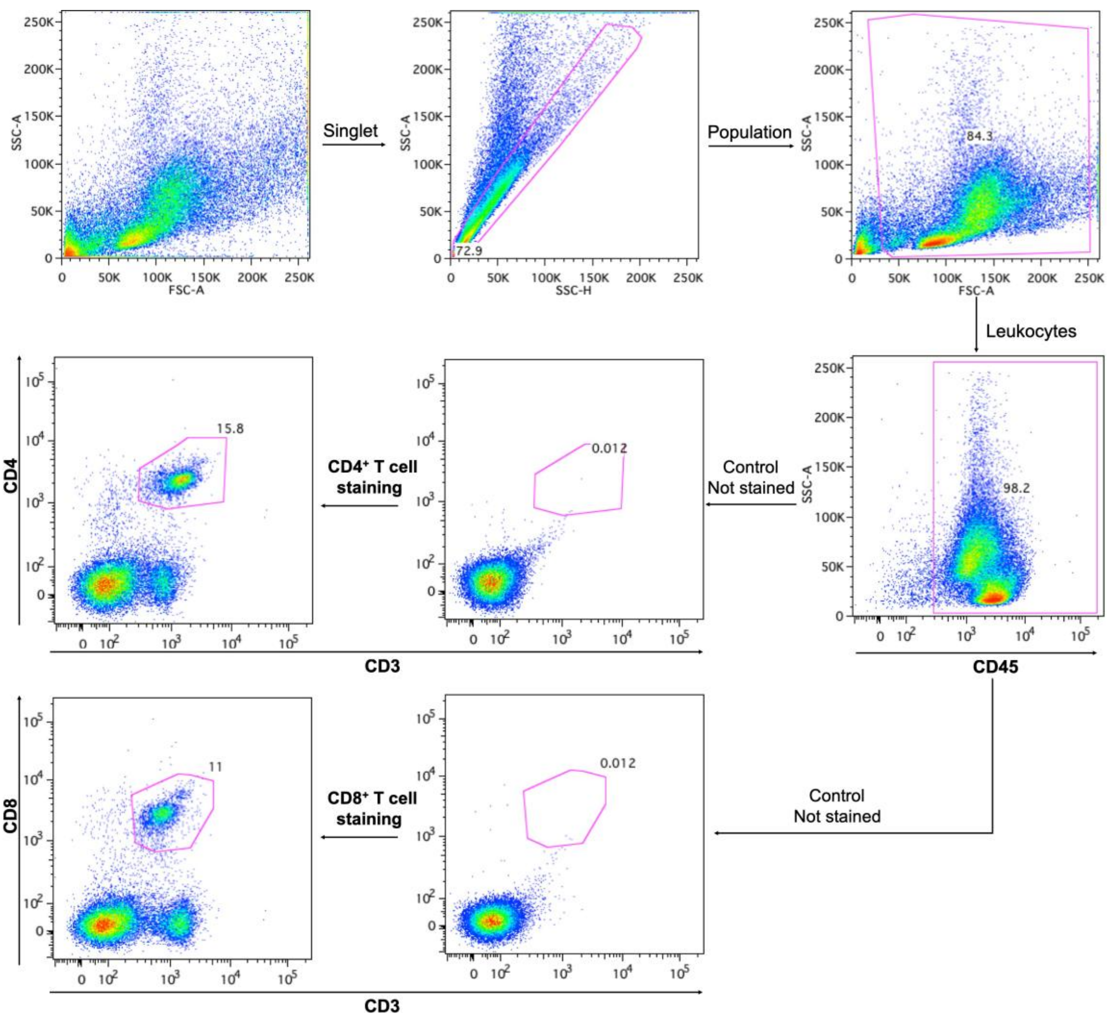

Suppl. Fig. 1: Gate strategy for CD4$^+$ and CD8$^+$ T-cells using the antibodies described on Suppl. Table 1. First, we selected the singlet cells and next a gate was drawn to exclude the debris. The leukocytes were selected based on CD45$^+$ staining and T-cells were selected according the double staining for CD3$^+$ and CD4$^+$ or CD8$^+$.

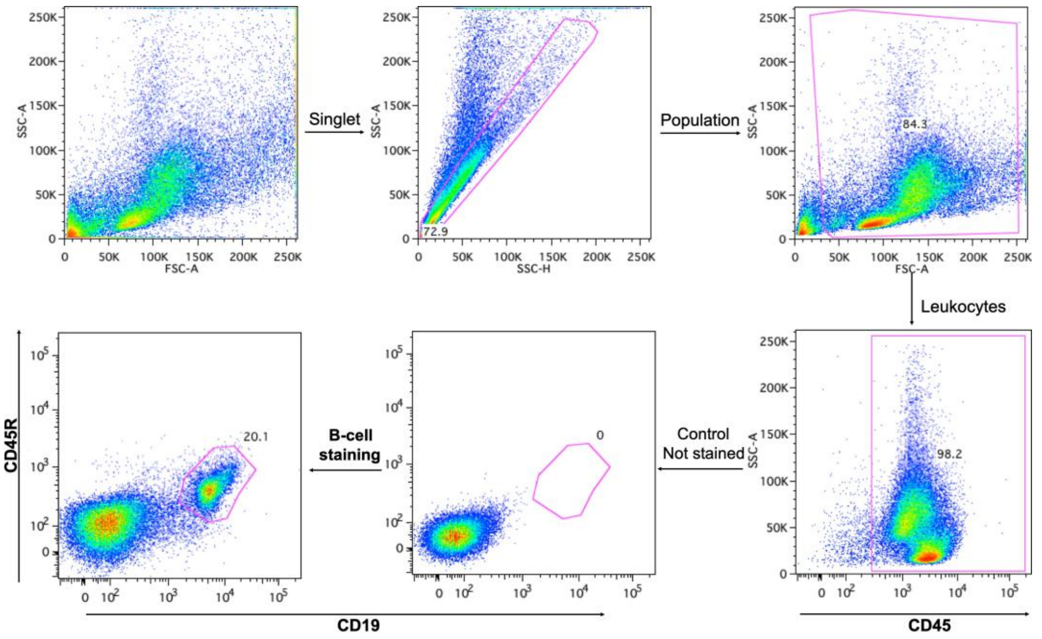

Suppl. Fig. 2: Gate strategy for B-cells using the antibodies described on Suppl. Table 1. First, we selected the singlet cells, and next a gate was drawn to exclude the debris. The leukocytes were selected based on CD45^{+} staining and B-cells were selected according the double staining for CD19^{+} and CD44R^{+}.

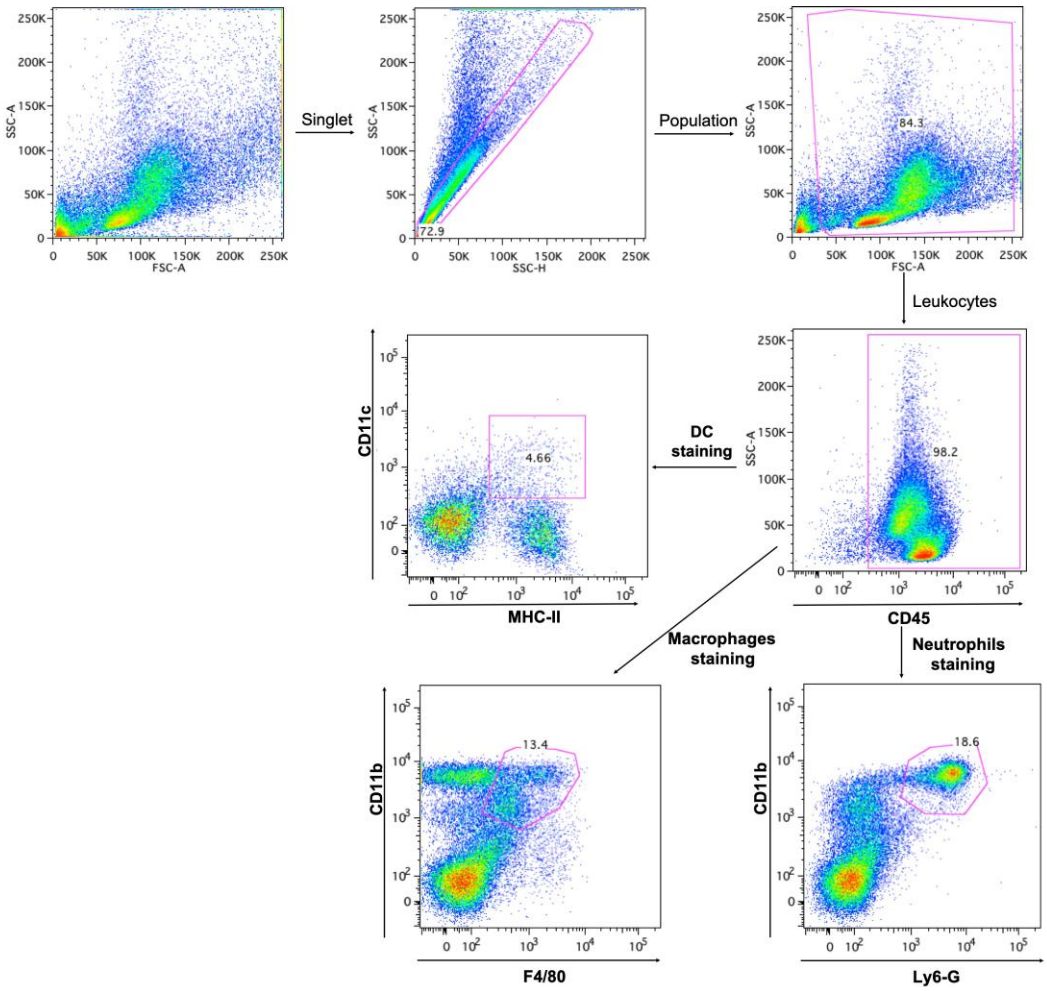

Suppl. Fig. 3: Gate strategy for myeloid cells using the antibodies described on Suppl. Table 1. First, we selected the singlet cells, and next a gate was drawn to exclude the debris. The

leukocytes were selected based on $CD45^+$ staining and dendric cell (DC) was selected based on the double staining for $CD11c^+$ and $MHC\text{-}II^+$. Macrophages were selected from double positive population based on $CD11b^+$ and $F4/80^+$ markers and neutrophils from $CD11b^+$ and $Ly6\text{-}G^+$ double-stained population.

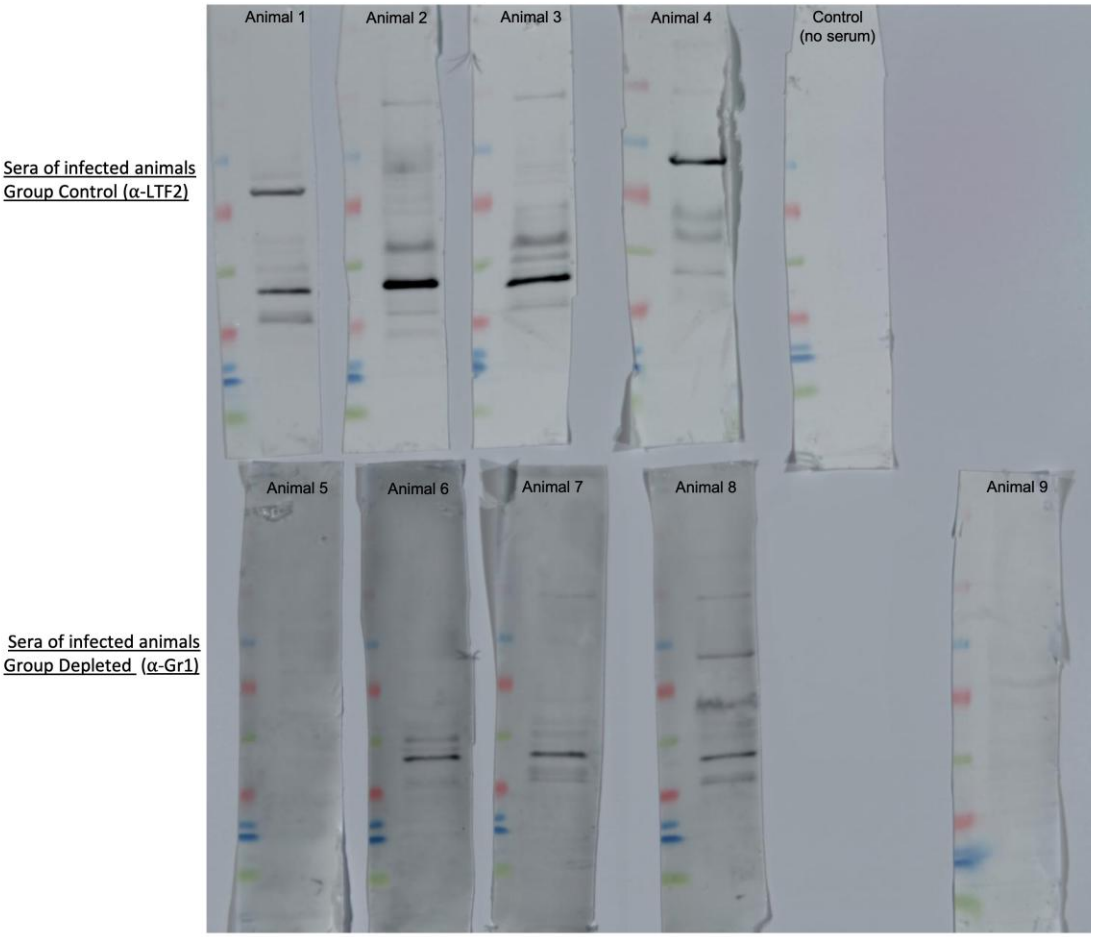

Suppl Fig. 4: Original file extract from ImageQuant LAS 500 (GE Healthcare ®). Ladder and the F. pedrosoi proteins were loaded in two SDS-PAGE gels. We ran both gels simultaneously and transferred to a nitrocellulose membrane, which was latter cut to contain one ladder and one sample per strip. After blocking, we incubated each strip with sera from a single animal, previously infected with *F. pedrosoi* for 14 days. All strips were then incubated with peroxidase-conjugated secondary antibody Positive signals were detected by enhanced chemiluminescence (SuperSignal West Pico, Pierce). All the strips were then developed simultaneously, using the same time exposure, so the bands could be further analyzed by the ImageJ software. Animals from control group (α-LTF2) showed higher amount of antibodies specific to *F. pedrosoi* proteins, when compared to the neutrophil depleted group (α-Gr1).
